# PREVALENCE OF SEXUAL AND GENDER MINORITY (SGM)–FRIENDLY POLICIES IN LONG-TERM CARE FACILITIES IN MINNESOTA

**DOI:** 10.1093/geroni/igae098.1613

**Published:** 2024-12-31

**Authors:** Tetyana Shippee, Rajean Moone, Jason Flatt, Ziwei Zhang, Morgan Wright, Nidhi Kohli, B R Simon Rosser

**Affiliations:** University of Minnesota School Of Public Health, Minneapolis, Minnesota, United States; University of Minnesota, St. Paul, Minnesota, United States; University of Nevada Las Vegas, Las Vegas, Nevada, United States; University of Minnesota, Minneapolis, Minnesota, United States; University of Minnesota, Minneapolis, Minnesota, United States; University of Minnesota, Minneapolis, Minnesota, United States; University of Minnesota, Minneapolis, Minnesota, United States

## Abstract

Sexual and gender minority (SGM) older adults in the U.S. face increased risk of Alzheimer’s disease and related dementias (AD/ADRD) and are more likely to utilize long-term services and supports (LTSS) compared to their cisgender, heterosexual peers. Despite these disparities, the prevalence of SGM-affirmative policies within LTSS settings remains understudied. This study aimed to comprehensively analyze the prevalence of SGM-affirmative policies in LTSS facilities in Minnesota. These include policies in human resources, marketing, training, governance; anti-discrimination policies guiding culturally responsive care, and resident sexual orientation and gender identity (SOGI) data collection. A policy analysis was conducted across 166 nursing homes and 267 assisted living facilities in Minnesota. Policies were assessed for their explicit inclusion of SGM-affirmative measures in various domains, and mean scores were calculated to determine the overall prevalence of such policies. The mean score of SGM-friendly policies across nursing homes and assisted living communities was 11.30. Interestingly, no significant disparities were found between ALFs and nursing homes or rural and urban facilities regarding the presence of SGM-friendly policies. However, using latent class analysis, we found that not for profit facilities and non-religious facilities were more likely to belong to a low performance group vs. high performance compared to their counterparts. Our results show the need for SGM-inclusive policies within LTSS settings. While overall prevalence was moderate, variations based on facility characteristics highlight opportunities for targeted interventions. Future efforts should focus on promoting SGM-affirmative policies across all LTSS facilities to ensure equitable and inclusive care for SGM older adults.

